# The sleep physiology of nightmares in veterans with psychological trauma: Evaluation of a dominant model using participant-applied electroencephalography in the home environment

**DOI:** 10.1111/jsr.13639

**Published:** 2022-05-29

**Authors:** Anne Richards, Steven H. Woodward, David Paul G. Baquirin, Leslie M. Yack, Thomas J. Metzler, Nikhila S. Udupa, Emily J. Staggs, Thomas C. Neylan

**Affiliations:** University of California San Francisco, and San Francisco VA Healthcare System, San Francisco, California, USA

**Keywords:** electroencephalography, heartrate, nightmares, posttraumatic stress disorder, rapid eye movement sleep, veterans

## Abstract

Nightmares are a core feature of posttraumatic stress disorder, are poorly understood, and are associated with serious negative outcomes. Their biology has been difficult to study, and the feasibility of capturing them in the naturalistic home environment has been poor. This said, the published research and dominant scientific model has focused on nightmares as a manifestation of noradrenergic hyperarousal during rapid eye movement sleep. The current study used at-home, participant-applied devices to measure nightmare physiology in posttraumatic stress disorder treatment-seeking veterans, by examining heartrate measures as indicators of noradrenergic tone, and sleep-stage characteristics and stability in the sleep preceding time-stamped nightmare awakenings. Our data indicate the high feasibility of participant-administered, at-home measurement, and showed an unexpected stability of -rapid eye movement sleep along with no evidence of heartrate elevations in sleep preceding nightmare awakenings. Altogether, these data highlight new opportunities for the study of nightmares while questioning the sufficiency of dominant models, which to date are largely theoretically based.

## 1 | INTRODUCTION

Nightmares are a hallmark feature of posttraumatic stress disorder (PTSD; Neylan et al., 1998), associated with subjective distress and serious negative outcomes, including suicide (Sandman et al., 2017). They are poorly understood because they are challenging to study: their occurrence can only be asserted after the fact in wake self-reports and they occur rarely in the sleep laboratory (Woodward, Arsenault, Murray, & Bliwise, 2000). While progress has been made in nightmare psychotherapies, effectiveness of and access to these is still wanting, and biological treatments remain unsatisfactory (Morgenthaler et al., 2018). Understanding the physiology underlying nightmares will help us develop targeted treatments to alleviate distress in frequent nightmare sufferers.

The prevailing model of PTSD nightmare physiology is that the noradrenaline-producing locus coeruleus is inappropriately active during rapid eye movement (REM) sleep in PTSD, resulting in REM fragmentation and defective sleep-dependent processing of emotional (trauma) information and eventually in distressing awakenings (Richards, Kanady, & Neylan, 2020). While this overall model is highly plausible, it remains to be demonstrated by empirical data. In fact, the two published reports of which we are aware that measured heartrate (HR), a proxy measure of noradrenergic activity, preceding reported nightmare-related awakenings surprisingly do *not indicate* elevated HRs prior to arousal (Phelps et al., 2018; Woodward, Michell, & Santerre, 2017). However, these HR measures may have been analysed with insufficient temporal resolution to adequately identify HR markers of noradrenergic arousal in the minutes prior to awakenings. Furthermore, no studies of which we are aware systematically measured REM sleep disruption, either by brief awakenings or transitions to other sleep stages thought less critical for emotion processing, prior to nightmare-related awakenings. Finally, the REM-sleep-focused model of trauma nightmares is undermined by evidence that PTSD nightmares occur in both REM and non-REM (NREM) sleep, such that the role of slee-pstage disruption and noradrenergic tone in both REM and NREM sleep needs to be considered (Richards et al., 2020).

The current analysis is based on data from a pilot study of at-home sleep measurement of trauma-related sleep disturbances. Our overall objective was to assess the feasibility of using participant-applied devices to study nightmare physiology in an ecologically valid environment. Our specific objectives with respect to nightmare biology were to examine HR and sleep-stage stability in the 10 min preceding nightmare awakenings. To capture even small increases in HR indicative of heightened noradrenergic activity, we hypothesized that there would be a statistically significant increase in HR in sleep preceding a nightmare arousal, without strict requirements such as beat-per-minute (bpm) change or tachycardia (100 bpm). In line with the predominant model of nightmare biology described above, we also hypothesized that pre-wake sleep, especially REM sleep, would be marked by instability including frequent brief awakenings and/or frequent epoch-to-epoch sleep-stage transitions.

## 2 | METHODS

Twenty-four US military veterans enrolled in a Veterans Affairs PTSD clinic and reporting trauma-related nightmares and/or nightmare enactment at least once per week were enrolled in a pilot study of at-home measurement of sleep using a sleep diary mobile app developed by the research team, a headband electroencephalography (EEG) device, and wrist actigraphy with event marker (Motionlogger MicroWatch; Ambulatory Monitoring, Ardsley, NY, USA). The Pittsburgh Sleep Quality Index-Addendum for PTSD (PSQI-A; Insana, Hall, Buysse, & Germain, 2013), modified in the study to ascertain frequency of nightmares occurring at least once weekly, was utilized to assess eligibility for the nightmare criterion. The only exclusions for enrollment were an inability to adhere to sleep diary instructions or to wear or use the actigraph. Participants completed 1–3 weeks of initial assessments using the diary and actigraph. Participants were instructed to compress the event marker immediately upon awakening from a distressing dream. The definition of a distressing dream as a sleep experience involving distressing imagery and explicitly recallable events was emphasized, to distinguish these from fearful awakenings without dream recall (Mellman, Kulick-Bell, Ashlock, & Nolan, 1995; Woodward et al., 2017). Participants who demonstrated adherence to sleep diary instructions, and who indicated at least one nightmare awakening weekly per actigraphy, were invited to participate in EEG assessments. The self-applied frontopolar EEG device, X8 Sleep Profiler-PSG2 (Ambulatory Monitoring) was used to collect overnight sleep recordings. Three frontopolar snap electrodes sampled at 256 Hz from approximate locations AF7, AF8 and Fpz generated outputs for EEG, electrooculography and electromyography. Forehead pulse rate was recorded at 100 Hz and calculated at 1 Hz using an optical sensor measuring photoplethysmography, as a proxy measure of HR. A validated automatic sleep-staging algorithm was initially used for sleep staging (Levendowski et al., 2017). Stages were determined based on the thresholds and ratios interdependent on power spectral density, ocular activity, micro and cortical arousals, and specific waveform characteristics such as sleep spindles. Data were subsequently reviewed and edited according to AASM scoring rules (Berry et al., 2018) epoch-by-epoch for the 10 min preceding a nightmare awakening by LY, a registered polysomnography technologist with extensive experience using the Sleep Profiler. Independent research indicates that despite some discrepancies due to limited topographical distribution of electrodes, there is strong agreement between visual scoring of recordings using Sleep Profiler and standard polysomnography (Lucey et al., 2016). The average pulse rate for each 30-s epoch was used to measure HR in the 10 min (20 epochs) prior to awakening. Clock time on the actigraph and Sleep Profiler were synchronized prior to delivery to participants. All actigraphy-marked awakenings were confirmed by concurrent awakenings on the EEG device. Classification of a nightmare awakening as a REM or NREM sleep awakening was based on the visual score of the last stage of sleep prior to the event marking. All study procedures were approved by human subjects research review committees at UCSF and the San Francisco VA.

### 2.1 | Statistical analyses

To examine hypothesis 1, regarding HR change over the 10 min (20 visually scored sleep epochs) preceding a reported nightmare awakening event, we performed a linear mixed-model regression, considering 20 repeated measures of HR within event and multiple reported events within subjects. To examine hypothesis 2, examining sleep-stage instability in REM and NREM sleep awakenings, we performed a Poisson regression with standard errors adjusted for clustering within subject (Hilbe, 2014).

## 3 | RESULTS

**FIGURE 1:**
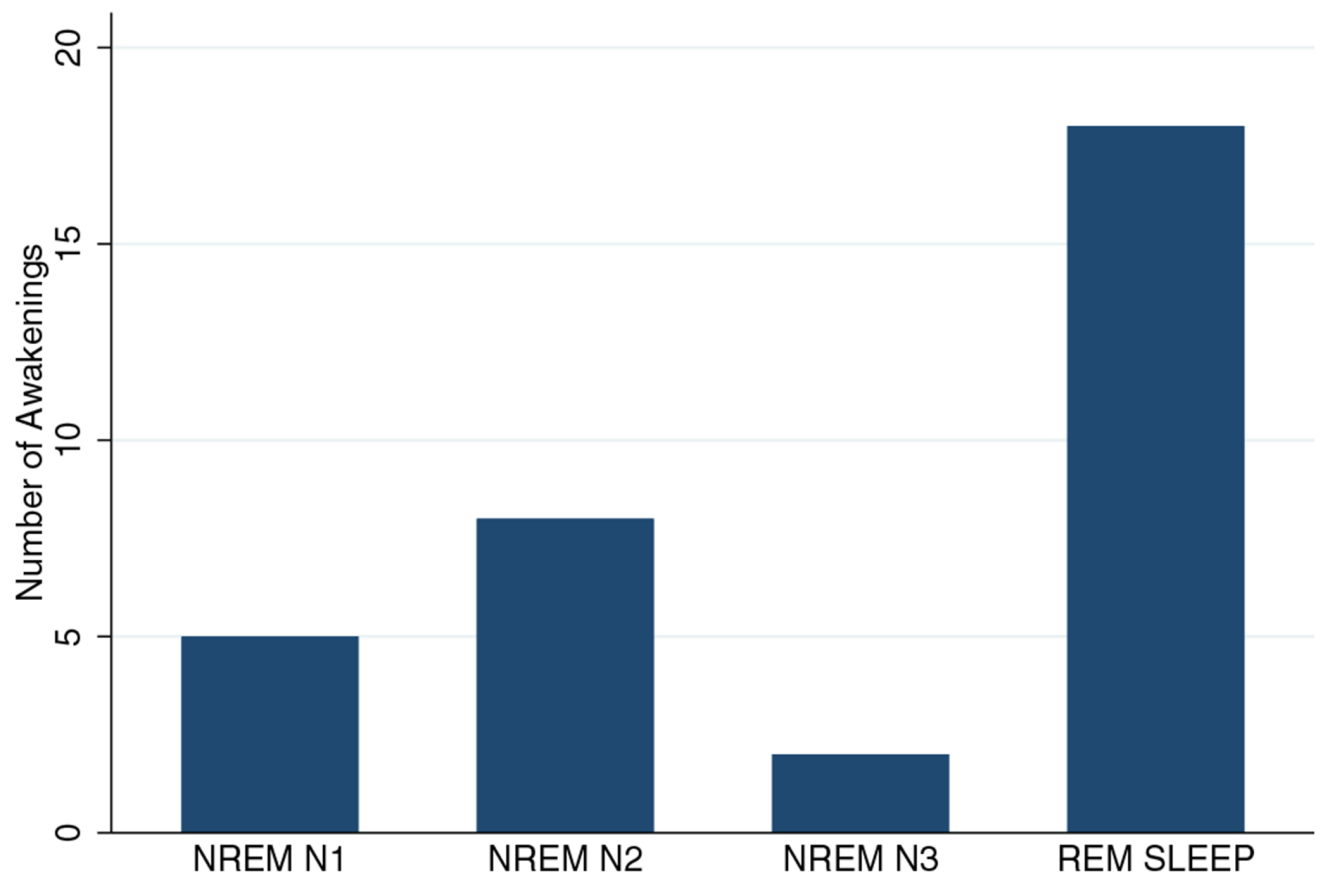
Sleep stage preceding nightmare awakenings (*N* = 33 events)

**FIGURE 2:**
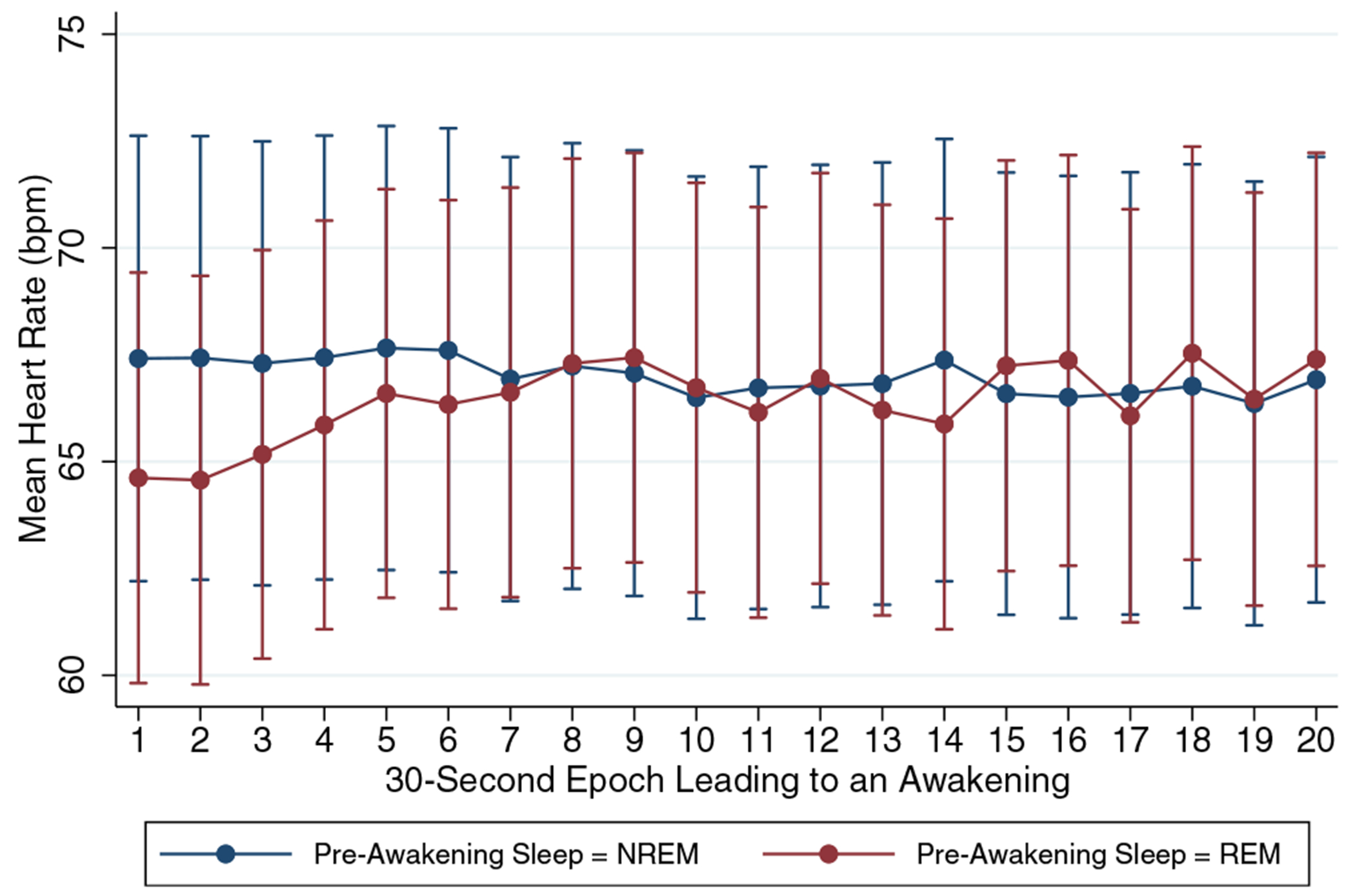
Mean heartrate (HR) in epochs leading to nightmare awakenings

Of 24 participants in Part 1 of the study, 11 participants were eligible for Part 2 based on frequency and adherence criteria. Data for one subject were rejected due to poor quality, yielding 33 nightmare events for analysis from 10 participants. Amongst these participants, three (30%) were female and the mean age was 46.7 years (SD 15.37). While most participants contributed 1–2 nightmare events during 6–9 nights of data collection, one participant provided data for 9 events (median number of events per participant = 2, modal number of events = 1). Eighteen (55%) nightmare awakenings were preceded by REM sleep, while 15 (45%) were preceded by various stages of NREM sleep (Figure 1). For hypothesis 1, mixed-model analysis demonstrated a significant increase in HR across the 10 min prior to awakening for REM sleep awakenings (*z* = 3.77, *p* < 0.001), but not for NREM sleep awakenings (*z* = −1.66, *p* = 0.096). However, HRs were in the normal range for resting wake, and HR changes could be considered trivial and non-clinically significant (Figure 2). With respect to hypothesis 2, counts of sleep-stage transitions in the 10 min prior to awakening demonstrated that REM sleep was in fact stable, demonstrating a mean of 1.2 transitions (median = 0; IQR: 0–2) prior to arousal. In contrast, NREM sleep was preceded by a relatively high and significantly different frequency of sleep-stage transitions (mean = 4.9, median = 4; interquartile range [IQR]: 3–6; Figure 3; *z* for difference = 3.60, *p* < 0.001).

Given the high number of NREM awakenings and unexpected findings with respect to REM sleep transitions, we performed a post hoc examination of pre-wake EEG data as well as survey data to better characterize subjective nightmare characteristics and associated sleep. Sleep terrors occur out of N3 sleep, typically happen in the first third of the night, are associated with marked autonomic arousal, and lead to partial awakenings in which individuals are confused, disoriented and only partially responsive. These may also be accompanied by brief dream-like mentation in adults (Oudiette et al., 2009). In the absence of data to distinguish between more complex narratives and brief, imagery-filled experiences, other evidence that NREM awakenings were sleep terrors might be informative. For both REM and NREM N1, N2 and N3 nightmare reports, we tabulated clock time of awakening, lag time between EEG awakening and actigraphy event reporting by the participant, the third of the participant’s sleep period in which the event occurred, percentage of N3 sleep in the 20 epochs pre-wake, and sleep terror score on the associated participant’s PSQI-A (with higher scores indicating more frequent sleep terrors; Table 1). Furthermore, to explore whether any NREM awakenings could be alternatively classified as REM sleep awakenings, we reported the percentage of epochs scored as REM sleep in the 20 epochs pre-wake. Inconsistent with a sleep terror designation for NREM events, this tabulation indicates that NREM awakenings occurred across the night, that N3 awakenings occurred in the second third of the night, and that all participants awakening from N3 sleep hit the event marker within 1 min of awakening. Furthermore, the nightmares reported by participants endorsing frequent sleep terrors (2 per week, score of 3) on the PSQI-A occurred after REM sleep awakenings (events #3, #4 and #8) or after sleep rich in REM epochs (event #20). The one awakening occurring out of fairly persistent N3 sleep (event #33) occurred in an individual without self-reported sleep terrors in the prior month. With respect to REM sleep percentage of pre-wake sleep, we observed that four events (#20, #22, #23 and #27) scored as NREM awakenings using our a priori approach in fact contained a large proportion of REM sleep epochs prior to awakening. This alternative characterization of pre-wake sleep raises the proportion of REM sleep awakenings and slightly reduces the stability of pre-wake “REM” awakenings. Table data nonetheless show that a large majority of REM sleep awakenings, even with a revised categorization, were preceded exclusively by REM sleep epochs. Re-analysis of transition counts between REM and NREM sleep using this new categorization actually strengthened the finding that REM sleep events are associated with reduced transitions relative to NREM events.

## 4 | DISCUSSION

This study used a participant-applied multimodal assessment and demonstrated the feasibility of studying nightmare physiology in the home environment. This facilitates the collection of data from much larger samples and the examination of physiological contributors about which current knowledge is largely theoretical.

These findings underscore the need to adjust the prevailing REM-sleep-focused narratives regarding PTSD nightmares. REM sleep accounts for ~20%–25% of sleep in normal sleepers (Carskadon & Dement, 2011), in PTSD (Richards et al., 2013), and in the 10 subjects studied here as per Sleep Profiler automated scoring (mean: 23.9%; SD 7%). Our finding that 55% of awakenings occurred out of REM sleep indicates that while nightmare awakenings may preferentially occur during REM sleep, this relationship is far from exclusive. In fact, Phelps et al. found that most nightmare awakenings occurred out of NREM sleep (Phelps et al., 2018).

Our data also show that REM sleep preceding nightmare awakenings is in fact quite unperturbed by transitions to and from other stages or epochs scored as wake, and that HR is fairly stable with no signs of clinically significant elevations. In contrast, NREM sleep was marked by transitions between stages 1, 2 and/or 3 and REM as well as epochs of wake, although it was similarly notable for absence of HR instability or elevations. We are not aware of other reports that have systematically examined sleep-stage transitions immediately preceding awakening from nightmares, despite the assumption that the sleep process is disrupted. In the absence of HR elevations during sleep, which are a chief indicator of adrenergic hyperarousal and the fight–flight process, the notion of nightmares as noradrenergic-hyperarousal-mediated phenomena becomes more tenuous. Woodward and colleagues have proposed there is a survival benefit to less aroused sleep in stressful environments (Woodward et al., 2017). Mellman and colleagues have highlighted the Hobson model, proposing that REM sleep arousals may be precipitated by increased noradrenergic firing (Mellman et al., 1995). Given the integrity of REM sleep and the HR stability observed prior to nightmare awakenings here, it is possible that non-noradrenergic neurobiology dominates during nightmares themselves, but may eventually trigger noradrenergic-mediated awakenings. The finding that alpha-blocking drugs reduced nightmares and might reduce REM sleep disruptions appropriately honed our focus on the noradrenergic system during REM sleep (Taylor et al., 2008). Enhanced ability to measure nightmares may now better position us to explore other neurochemicals involved in nightmare generation. Examining the role of cannabinoid and arousal neurochemical (e.g. orexin) signalling is warranted. The function of memory systems in nightmare generation, recurrence and impact on daytime intrusions is also ripe for investigation.

Notwithstanding these findings, this pilot study has important limitations. Although the number of reported events was large relative to most reports on trauma nightmares, the sample size was small and one subject contributed a disproportionate number of events to the analysis. Statistical methods accounted for the clustering of events within participant; however, larger sample sizes are necessary for generalizable conclusions. Additionally, our post hoc examination of percent REM sleep indicates that four events scored as NREM awakenings in fact contained a large proportion of REM sleep epochs prior to awakening. The sleep stage immediately preceding the awakening was used to categorize pre-wake sleep type, simply because we have no information about when a nightmare may have started or stopped. Our a priori approach was the least biased and least arbitrary approach. However, depending on the duration of the nightmare, it is possible that some of those nightmares occurred predominantly during REM sleep. Also, this pilot study did not collect details about the subjective severity or content of the reported dreams. Nonetheless, all participants were selected for subjective high frequency (> once/week) of nightmares, such that these data likely provide a faithful reflection of the regular distressing dream experiences of individuals reporting frequent nightmares, including those described in epidemiological studies linking frequent nightmares to suicide. And while we did not collect detailed information about narrative complexity, our post hoc analyses provide evidence against the possibility that most NREM events were sleep terrors, rather than nightmares. Overall, however, they indicate that PTSD sleep is characterized by irregular sleep patterns and subjective perturbations that may be inconsistent with classical definitions.

In conclusion, advances in ambulatory methods hold great promise for advancing our knowledge of nightmares. This creates the opportunity to revise the tenacious but likely oversimplistic noradrenergic system and REM-sleep-focused model of PTSD nightmares. One can now imagine a horizon on which nightmare biomarkers are identified, allowing for a deeper probing of their generation and, with that, targeted treatment solutions.

## Figures and Tables

**FIGURE 3:**
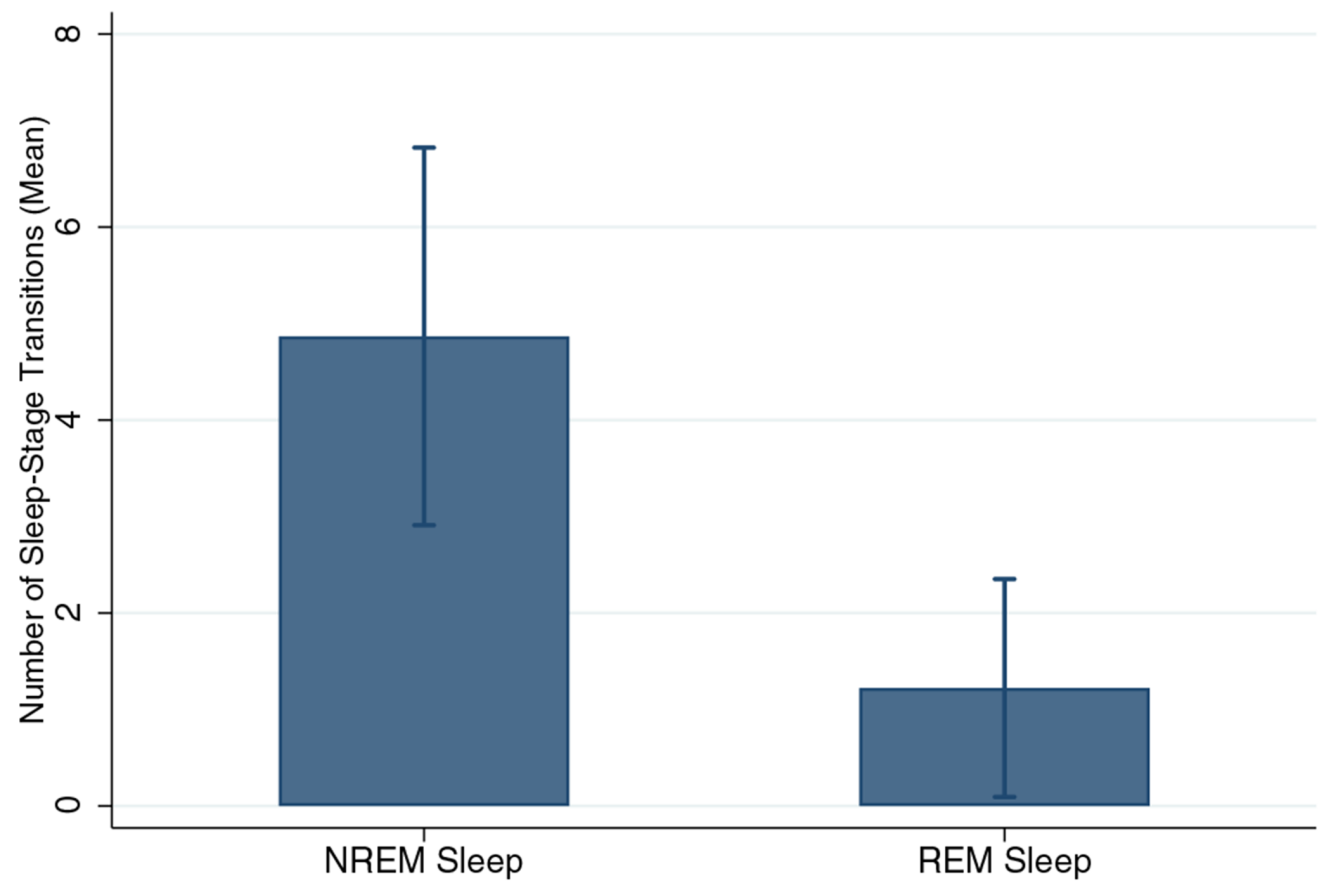
Sleep-stage transitions preceding rapid eye movement (REM) and non-REM (NREM) sleep awakenings

**TABLE 1.** Characteristics of pre-wake sleep and associations with PSQI-A sleep terror score for reported nightmare awakening events, including stage of sleep for epoch preceding awakening (REM or NREM N1, N2 or N3); clock time of awakening recorded on EEG device; lag time between EEG awakening and actigraphy event reporting by the participant; the third of the participant’s sleep period in which the event occurred; the percentage of REM sleep and N3 sleep in the 20 epochs preceding the awakening; and the PSQI-A sleep terror score of the participant reporting the event. Higher PSQI-A scores using the response options modified for our study indicate more frequent sleep terrors (0= not during the past month; 1 = less than once per week; 2 = once per week; 3 = twice per week; 4 = three or more times per week). REM = rapid eye movement sleep; NREM = non-rapid eye movement sleep; N1= stage 1 sleep; N2= stage 2 sleep; N3 = stage 3 sleep; EEG = electroencephalogram

Event number	Awakening type (NREM versus REM)	Wake clock time per EEG device	Lag time - EEG wake to actigraph event marking (min)	Third of participant’s night	Percent REM epochs in pre-wake sleep	Percent N3 epochs in pre-wake sleep	Self-reported frequency of sleep terrors; modified PSQI-A score
1	REM	05:53 hours	< 1	2	35%	0	1
2	REM	03:38 hours	< 1	1	80%	0	1
3	REM	02:03 hours	< 1	2	100%	0	3
4	REM	03:31 hours	< 1	1	100%	0	3
5	REM	03:07 hours	< 1	2	100%	0	0
6	REM	06:33 hours	< 1	2	100%	0	1
7	REM	03:08 hours	< 1	1	100%	0	1
8	REM	07:46 hours	< 1	3	100%	0	3
9	REM	01:21 hours	< 1	1	75%	0	1
10	REM	05:10 hours	< 1	3	90%	0	1
11	REM	05:46 hours	< 1	3	45%	0	1
12	REM	03:25 hours	< 1	2	100%	0	0
13	REM	05:06 hours	< 1	3	100%	0	0
14	REM	12:05 hours	< 1	1	100%	0	0
15	REM	23:44 hours	< 1	1	100%	0	0
16	REM	03:46 hours	< 1	2	100%	0	0
17	REM	05:41 hours	< 1	3	100%	0	0
18	REM	04:38 hours	< 1	3	75%	0	0
19	N1	05:59 hours	< 1	2	35%	0	1
20	N1	03:25 hours	1.5	3	65%	0	3
21	N1	22:41 hours	4.5	1	0	0	1
22	N1	01:25 hours	< 1	2	60%	0	1
23	N1	04:30 hours	< 1	3	95%	0	0
24	N2	05:37 hours	< 1	2	0	0	1
25	N2	03:08 hours	< 1	1	0	0	1
26	N2	03:17 hours	< 1	1	0	0	1
27	N2	05:30 hours	< 1	3	65%	0	1
28	N2	05:13 hours	< 1	2	0	20%	1
29	N2	03:08 hours	< 1	1	0	0	1
30	N2	02:19 hours	< 1	2	0	0	1
31	N2	02:27 hours	< 1	2	0	5%	1
32	N3	03:49 hours	< 1	2	0	5%	1
33	N3	02:26 hours	< 1	2	0	85%	0

Abbreviations: EEG, electroencephalography; NREM, non-rapid eye movement; PSQI-A, Pittsburgh Sleep Quality Index-Addendum; REM, rapid eye movement.

## Data Availability

The data that support the findings of this study are available from the corresponding author upon reasonable request.
